# War’s youngest victims: a descriptive cross-sectional study on injury distributions, severity patterns, and outcomes among paediatric trauma patients in Kharkiv, Ukraine

**DOI:** 10.1186/s13031-025-00694-w

**Published:** 2025-07-26

**Authors:** Filippa Sennersten, Safora Frogh, Gustav Falk, Mariia Matvieienko, Olha Karafulidi, Olha Konstantynovska, Yohan Robinson, Andreas Wladis, Denise Bäckström

**Affiliations:** 1 Department of Biomedical and clinical Sciences, Linköping University, Linköping, 581 83 Sweden; 2 Department of Leadership and Command & Control, Swedish Defence University, Karlstad, Sweden; 3 Karolinska Institutet, Stockholm, Sweden; 4 V.N. Karazin Kharkiv National University, Kharkiv, Ukraine; 5 Kharkiv City Clinical Hospital of Emergency Aid named by prof. O.I. Meshchaninov, Kharkiv, Ukraine; 6 Imperial College London, London, UK; 7 Department of Surgery, University of Gothenburg, Gothenburg, Sweden

**Keywords:** Paediatric trauma, War injuries, Injury severity, Blast trauma, Kharkiv, Ukraine, Rehabilitation

## Abstract

**Background:**

Paediatric trauma in conflict zones is a major public health concern, with children being highly susceptible to both immediate injuries and long-term disabilities. The Russian Invasion of Ukraine in February 2022 has significantly affected the civilian population, particularly children. However, despite reports highlighting the extent of paediatric casualties, empirical data on injury patterns among children in the ongoing conflict remains scarce.

**Objective:**

Describe the injury distributions, severity and outcomes of paediatric war-related trauma patients during the Russian invasion of Ukraine, using a hospital-based cohort from Kharkiv.

**Methods:**

This descriptive cross-sectional study examined 64 war-related paediatric trauma patients (0–17 years) admitted to two Kharkiv hospitals between February 2022 and November 2023. Injury mechanisms, severity (using Abbreviated Injury Scale and Injury Severity Score [ISS]), and clinical outcomes were assessed.

**Results:**

War-related injuries, including blast and shelling, accounted for 26% of all cases. Temporal analysis showed two peaks in injury incidence correlating with significant military events in the region. Analysis of the war-related injuries indicated that the most affected body regions included the upper extremities (31%), lower extremities (28%), and thorax (28%). The median ISS was 9, with over one-third (36%) of patients sustaining severe or critical injuries. Outcomes at discharge revealed a majority, 52%, achieved good recovery, followed by 41% with moderate disabilities. A smaller percentage (3%) faced severe disabilities, while 5% of the cases resulted in fatalities.

**Conclusions:**

This study offers new insights into paediatric trauma from modern warfare in a high-income setting, highlighting the occurrence of blast and shelling injuries, injury patterns overall similar to previous conflicts, and high disability rates at discharge. The findings underscore the need for comprehensive trauma care, including acute treatment and long-term rehabilitation, and can inform improvements in care protocols, resource allocation, and rehabilitation strategies.

## Background

Paediatric trauma is a major public health issue in conflict zones, with children being highly vulnerable to both immediate injuries and long-term health effects [1]. The Russian invasion of Ukraine in February 2022 has highlighted the severe impact of warfare on civilians, including children [2]. In December 2024, UNICEF reported that 20% of children in Ukraine require ongoing humanitarian support, with explosive weapons causing 87% of child casualties [3]. Despite these alarming statistics, no empirical data or peer-reviewed studies have been published on the distribution and severity of paediatric injuries during this conflict.

The epidemiology of paediatric trauma in other conflict zones, such as Afghanistan, Iraq, and Syria, reveal overall similar injury distributions to adults, including injuries to the extremities, head, abdomen and thorax [4, 5]. Existing literature does however highlight the prevalence of blast-related injuries in the paediatric population, which disproportionately affect children due to their physical vulnerability [4]. Children also have a slightly higher mortality rate, with burns and head injuries posing greater risks [4, 5]. However, previous studies in this domain have focused on conflicts in low- and middle-income countries, with limited healthcare infrastructure and resources.

Prior to the conflict, Ukraine operated a state-funded healthcare system with universal access, although regional disparities persisted [6, 7]. Child healthcare was delivered through a dual model, with roughly half of children treated by paediatricians while the other half by family physicians, which was supported by a network of specialised and non-specialised paediatric hospitals and polyclinics [6]. Before the invasion, Ukraine was also undertaking significant efforts to modernise its trauma care system, aiming to transition from a Soviet-era model to one aligned with Western standards [7]. Reforms initiated by the Ministry of Health focused on developing emergency medical services (EMS), introducing emergency medicine training programs, and establishing a national EMS dispatch system [8]. However, these improvements were still in early stages, and key components, such as standardised trauma protocols, integrated prehospital and hospital care, effective communication systems, and a national trauma registry, remained underdeveloped [9]. As a result, when the war began in February 2022, critical gaps in coordination, equipment, and rehabilitation services were further revealed.

The Kharkiv region, in northeastern Ukraine, near the border with Russia, has been one of the most heavily impacted areas since the invasion began in February 2022 [10]. As the war progressed, most military engagements concentrated on the front lines. However, Kharkiv’s geographical proximity to the border has continued to expose the region to indirect fire, including shelling and multiple launch rocket system attacks [11, 12]. Even with shifts in the intensity of hostilities, the persistent threat of missile strikes, and artillery fire has contributed to ongoing civilian casualties and widespread destruction.

This study aims to describe the injury distributions, severity and outcomes of paediatric trauma patients during the Russian invasion of Ukraine, using a hospital-based cohort from Kharkiv. By addressing the current lack of empirical data on paediatric injury patterns during the current conflict, the study seeks to provide important insights that may guide trauma care protocol development, long-term rehabilitation strategies for affected children, and resource allocation optimisation in ongoing and future conflicts.

## Methods

### Study design

This descriptive cross-sectional study aims to analyse the clinical characteristics, epidemiology, and outcomes of paediatric war-related trauma cases treated in hospitals across the Kharkiv region following the onset of the Russian invasion of Ukraine on February 24, 2022. The study adheres to the Strengthening the Reporting of Observational Studies in Epidemiology (STROBE) guidelines to ensure methodological and comprehensive reporting.

### Study setting

Before the full-scale invasion, the population of the Kharkiv region was approximately 2.6 million people [13]. Although statistics regarding the paediatric population are not readily available, the region’s child population has been previously reported to be around 426,000 children [14]. These children primarily received outpatient care from family physicians and paediatricians, with more complex cases referred to regional children’s hospitals for inpatient management [14].

This study was conducted at two tertiary-level public hospitals in the Kharkiv region: the Kharkiv City Clinical Hospital of Emergency Aid and the Kharkiv Regional Clinical Hospital for Children. Both facilities were designated as paediatric trauma centers before the onset of the Russian invasion and remained operational throughout the study period.

#### Prehospital care

Ukraine’s prehospital care system has remained functionally resilient during the ongoing armed conflict, although it has been affected by infrastructure damage and security concerns [15]. In Kharkiv and other conflict zones, emergency medical services (EMS) include state-controlled ambulance services, private volunteer groups, and NGO-supported initiatives. Since the onset of hostilities, EMS protocols have been adapted to incorporate tactical medicine principles and the use of protective gear for personnel.

The amount of emergency medical responses has reportedly remained unchanged [15], but the quality of prehospital care has been described as being highly variable [9]. While some patients receive timely and structured transport, others have limited or no access to prehospital interventions. Notably, these reports are not specific to the paediatric population, and there is limited data on how children have been managed during the prehospital phase.

#### Trauma care

Ukraine’s trauma system, both administrative and clinical, has long been under-resourced and outdated [9, 16]. Key limitations include lack of standardised trauma curriculum, absence of Advanced Trauma Life Support (ATLS)-equivalent training, and inadequate infrastructure. While the war has triggered some curricular adjustments in surgical and trauma training, systemic gaps persist [16].

For paediatric trauma, the situation is even more constrained. The war has led to an increase in war-related injuries that require practicing providers to transition and perform complex surgical interventions and provide comprehensive long-term care [17]. The need for specific paediatric trauma training pathways have increased in order to meet the acute needs of the injured children. Unfortunately, the clinical protocols for managing injured children remain poorly described in the literature and no formal trauma registries or outcome studies focusing on children in the Kharkiv region currently exist.

#### Rehabilitation services

Rehabilitation services for trauma patients, particularly children, are underdeveloped [9]. Reports suggest that much of the rehabilitation burden falls on families, who may lack access to structured physical or occupational therapy services. Children requiring specialised rehabilitation have reportedly also been referred to neighbouring countries for care. Overall capacity remains limited, and there is a lack of coordinated paediatric rehabilitation frameworks in the region [9, 14].

### Study population

The cohort comprised 64 paediatric patients aged 0–17 years admitted during the designated time period for war-related trauma injuries. Eligible patients were identified from hospital records using the following inclusion and exclusion criteria:

- **Inclusion Criteria**:
  - Patient age between 0 and 17 years.
  - Presence of isolated, associated, or combined trauma.
  - Admission to hospital within 24 h of injury, or transfer from a lower-level institution within 3 days of injury.
  - Trauma sustained from war-related mechanisms (e.g., blasts, shelling).
- **Exclusion Criteria**:
  - Patients aged over 18 years.
  - Unavailable medical records.
  - Pre-hospital fatalities.

All eligible cases from participating hospitals were included sequentially based on admission date to minimise selection bias.

### Variables

Data was extracted from hospital medical records by trained medical personnel, including local Ukrainian physicians and medical students, using a standardised data collection form to ensure consistency across sites. Variables collected included:

1. **Demographic and Medical History**:

- Age, gender, weight, height, and pre-existing medical conditions.

2. **Incident Details**:

- Date, mechanism of injury, and admission and discharge dates.

3. **Injury Characteristics**:

- Injury distribution by body region.
- Injury severity measured using the Abbreviated Injury Scale (AIS) and Injury Severity Score (ISS).

4. **Clinical Outcomes**:

- Length of hospital stay.
- Discharge status (e.g., home, rehabilitation centre, further hospitalisation).
- Functional status at discharge.

### Data sources and measurement

Hospital records served as the primary data source. Injury severity was calculated using the AIS and ISS, which are internationally recognised tools for quantifying trauma severity [18]. The ISS scores were calculated for each patient, categorising injuries as mild (1–8), moderate (9–15), severe (16–24), or critical (≥ 25). Functional outcomes were determined based on clinician-recorded discharge summaries using qualitative descriptors and supported by clinical data. Functional outcomes were categorised as either good recovery, moderate disability, severe disability, or fatality.

### Statistical methods

Descriptive statistics were employed to summarise patient demographics, injury mechanisms, and outcomes. Continuous variables were reported as means and medians. Categorical variables were presented as frequencies and percentages. Comparative analyses included Fisher’s exact tests to evaluate associations between demographic variables (e.g., age, gender) and injury characteristics. Statistical significance was set at *p* < 0.05. Statistical analyses were performed using Stata version 18.0 (StataCorp, College Station, Texas 77845 USA).

### Bias

This study is subject to biases common in retrospective observational research, especially in conflict zones. Survivor bias likely led to underestimation of injury severity and mortality, as pre-hospital fatalities were excluded. Selection bias may have occurred due to inclusion of only two regional hospitals, excluding milder injuries or cases from other facilities. Referral bias may also be present if these hospitals disproportionately received more severe cases due to their trauma designation.

Information bias was introduced by the potential of incomplete or inconsistent documentation and record loss during the conflict. Temporal bias is possible due to changes in injury trends over the 22-month study period. Reporting bias may affect functional outcomes, as standardised disability measures were not uniformly used at discharge. Finally, potential confounding variables such as socioeconomic background or pre-existing health conditions were not consistently captured in the data, which may influence interpretation of disability or mortality outcomes.

These biases were all considered in the interpretation of results and presented in the discussion, in accordance with the STROBE guidelines.

## Results

During the study period, a total of 64 war-related paediatric trauma cases were documented, encompassing shelling and blast injuries. No gunshot wounds were recorded. The group’s demographic characteristics, injury mechanisms, injury characteristics, and outcomes are described below.

### Demographic data and medical history

Of the 64 war-related paediatric trauma patients, 42 were males (66%) and 22 females (34%). Sex did not show a statistically significant association with any injury type, and there was no statistically significant association between sex and ISS group (all *p* > 0.05).

The mean age was similar between sexes, with boys averaging 10.3 years and girls 11.8 years. For statistical analyses, age groups 0–5 years old, 6–11 years old, and 12–17 years old, were formed. Only “Face Injury” vs. Age 12–17 group showed a significant association (*P* = 0.044), although clinically irrelevant. All other injury types had no significant association (*p* > 0.05). None of the age groups show a significant association with ISS groups (all *p* > 0.05).

Detailed information on injury distribution and severity by sex and age group is provided in Tables 1 and 2. *P*-values concerning all Fisher’s exact test results for sex and age group comparisons can be found in Table 3, Appendix.

Only two patients had recorded prior medical conditions: asthma (*N* = 1) and diabetes (*N*= 1).

**Table 1:** Injury distribution by sex, age groups, and body regions. Note: total exceeds *N* = 64 due to multiple injuries per patient across body regions

Injury Type	Total Cases, *N*	Males, *N* (%)	Females, *N* (%)	Age 0–5 years, *N* (%)	Age 6–11 years, *N* (%)	Age 12–17 years, *N* (%)
**Head**	13	6 (46%)	7 (54%)	3 (23%)	3 (23%)	7 (54%)
**Face**	10	5 (50%)	5(50%)	3 (30%)	5 (50%)	2 (20%)
**Cervical**	1	1(100%)	0 (0%)	1(100%)	0 (0%)	0 (0%)
**Thorax**	18	12 (67%)	6 (33%)	1 (6%)	5 (28%)	12 (67%)
**Lumbar**	1	0 (0%)	1 (100%)	0 (0%)	1 (100%)	0 (0%)
**Abdomen**	7	5 (71%)	2 (29%)	1(14%)	2 (29%)	4 (57%)
**Upper Extremity**	20	13 (65%)	7 (35%)	2 (10%)	5 (25%)	13 (65%)
**Lower Extremity**	18	12 (67%)	6 (33%)	1 (6%)	8 (44%)	9 (50%)
**Burns**	4	3(75%)	1 (25%)	2 (50%)	0 (0%)	2 (50%)
**External**	7	3 (43%)	4 (57%)	1 (14%)	3 (43%)	3 (43%)

N = numbers

**Table 2:** Injury severity by sex and age groups

Injury Severity Score	Total Cases, *N*	Males, *N* (%)	Females, *N* (%)	Age 0–5 years, *N* (%)	Age 6–11 years, *N* (%)	Age 12–17 years, *N* (%)
**ISS (1–8)**	22	15 (68%)	7 (32%)	4 (18%)	8 (36%)	10 (46%)
**ISS (9–15)**	19	11 (58%)	8 (42%)	4 (21%)	7 (37%)	8 (42%)
**ISS (16–24)**	14	11 (79%)	3 (21%)	2 (14%)	4 (29%)	8 (57%)
**ISS (> 25)**	9	5 (56%)	4 (44%)	1 (11%)	1 (11%)	7 (78%)

N = numbers

**Table 3:** Fisher’s exact test results for sex and age group comparisons

Category	Sex (*p*)	Age 0–5 years (*p*)	Age 6–11 years (*p*)	Age 12–17 years (*p*)
**INJURY TYPES**				
**Head**	0.362	0.411	0.539	1.000
**Face**	0.509	0.175	0.285	0.044*
**Cervical**	1.000	0.152	1.000	0.475
**Thorax**	0.606	0.293	0.784	0.204
**Lumbar**	0.394	1.000	0.323	0.475
**Abdomen**	0.701	1.000	1.000	1.000
**Upper Extremity**	0.799	0.729	0.594	0.316
**Lower Extremity**	0.606	0.293	0.269	1.000
**Burns**	1.000	0.108	0.301	1.000
**External**	0.429	1.000	0.678	0.705
**INJURY SEVERITY SCORE**				
**ISS (1–8)**	0.790	1.000	0.577	0.600
**ISS (9–15)**	0.406	0.719	0.565	0.415
**ISS (16–24)**	0.346	1.000	1.000	0.765
**ISS (> 25)**	0.480	1.000	0.252	0.150

p = *p*-value

* = statistically significant

### Incident details

**Fig. 1:**
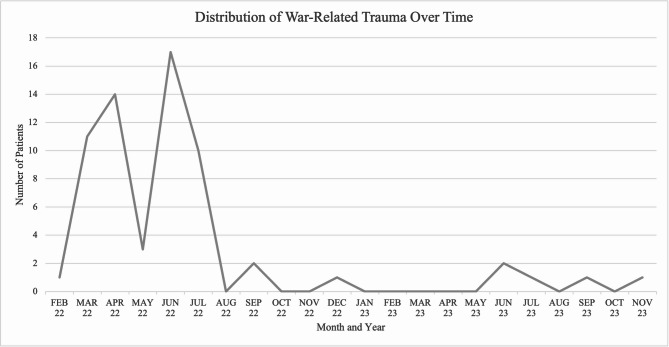
Distribution of war-related trauma over time

The occurrence of war-related paediatric injuries varied over time, with two peaks, one right after the invasion and the other in June 2022 (*N* = 17) when Kharkiv region (40% of the territory) was reclaimed by the Ukrainian army. The distribution of war-related trauma cases over time is illustrated in Fig. 1.

### Injury characteristics

**Fig. 2:**
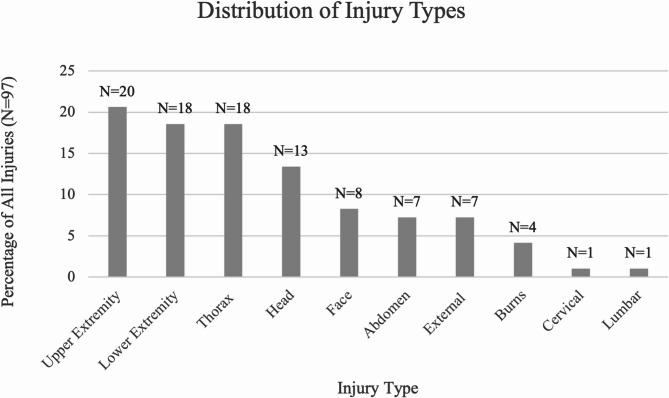
Distribution of injury types. External injuries refer to abrasions, avulsion and lacerations

War-related injuries were widely spread across different body regions, with a significant number involving the head, thorax, and extremities, as seen in Fig. 2. Several patients (*N* = 25, 39%) suffered from injuries involving multiple body regions.

Head injuries were observed in 13 patients. The injuries ranged in severity, with subdural haemorrhages (*N* = 4) and concussions (*N* = 3) being the most common. Additionally, there were open wounds of the head (*N* = 3), as well as isolated cases of epidural haemorrhage, subarachnoid haemorrhage, and soft tissue injuries. Facial injuries were observed in eight patients, with the majority involving soft tissue damage (*N* = 7), and one eye injury.

A single case of cervical injury was recorded, involving an open wound of the neck, and one patient suffered an unspecified lumbar injury.

Thoracic injuries were recorded in 18 patients. Among these, thoracic fractures (*N* = 4) and thoracic cord injuries (*N* = 2) were reported. The majority of thoracic injuries, however, were classified as unspecified (*N* = 12).

Abdominal trauma was recorded in seven patients, with injuries distributed across multiple organs. Patients suffered from bladder injury (*N* = 1), liver lacerations (*N* = 2), spleen laceration (*N* = 1), and other unspecified abdominal injuries (*N* = 3).

Extremity injuries were common among the paediatric trauma cases in this subgroup with war related injuries, with 20 patients sustaining injuries to the upper limbs and 18 patients to the lower limbs. The majority of upper extremity injuries were penetrating wounds (*N* = 11). Additionally, fractures were common, with cases involving the forearm/wrist (*N* = 2), hand (*N* = 1), humerus (*N* = 1), and clavicle (*N* = 1), alongside a case of compartment syndrome. Lower extremity injuries were similarly distributed, with penetrating trauma (*N* = 10) being the most frequent, followed by femur fractures (*N* = 2), foot fractures (*N* = 2), and pelvic fractures (*N* = 2).

Seven patients presented with external injuries involving abrasions (*N* = 4), avulsion (*N* = 1), and lacerations (*N* = 2). Thermal burns were observed in four patients.

### Injury severity

**Fig. 3:**
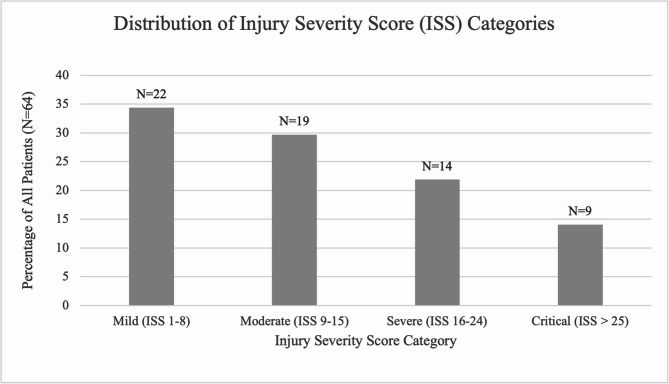
Distribution of Injury Severity Score (ISS) categories

The distribution of ISS values among the paediatric trauma patients with war related injuries in this study reflects a range of injury severities, as detailed in Fig. 3.

The median Injury Severity Score (ISS) was 9. The majority of patients (64%) sustained injuries categorised as mild (ISS 1–8) or moderate (ISS 9–15), and often involved isolated injuries. A proportion of patients (36%) experienced severe (ISS 16–24) or critical (ISS ≥ 25) injuries. These cases were associated with complex trauma involving multiple body regions. Notably, the highest ISS scores (ISS 25–50) were observed in nine patients.

### Clinical outcomes

Time of hospitalisation ranged from 1 to 44 days with a mean hospital stay of 8.5 days, and a median of 5 days.

Regarding patient outcomes, 33 patients (52%) had a good recovery, while 26 patients (41%) were discharged with moderate disabilities. Severe disabilities were noted in two patients (3%), and there were three fatalities (5%).

Discharge destinations included home for 29 patients (45%), rehabilitation centres for ten patients (16%), and other hospital wards for 19 patients (30%). Two patients required transfer to intensive care units with a higher level of care, while three patients were transferred to the morgue.

## Discussion

The study provides a unique and comprehensive characterisation of injury patterns among children exposed to war-related trauma in the Kharkiv region following the Russian invasion of Ukraine in February 2022. It offers novel insights into conflict-related injuries among children in a modern, high-income European setting. Key findings include the prominence of blast and shelling injuries, similarities in injury distributions to previous conflicts, and the substantial proportion of patients discharged with disabilities. These findings are important first steps in understanding the war’s effect on the civilian paediatric population and improving trauma care, resource allocation, and rehabilitation planning.

One main finding of the article is the predominance of blast and shelling injuries, as all documented injuries were caused by these mechanisms of injury. Blast and shelling injuries were also grouped as it was challenging for the data collectors to distinguish between the two. Notably, there were no reported gunshot, landmine, and unexploded ordnance (UXO) injuries among admitted children, which is likely attributed to evacuations of civilians from the front-line. Instead, injuries in cities appear to result primarily from indirect fire, reflecting the broader impact of the conflict beyond active combat zones [12].

The temporal distribution of injuries reflects the dynamic nature of the conflict and is illustrated in Fig. 2. Injury rates peaked during the early months of the invasion, particularly in March–April 2022 and June–July 2022, aligning with the occupation and liberation of the region. The first peak corresponds to active hostilities when the front line was closest to Kharkiv, allowing the use of multiple launch rocket systems by Russian forces. The subsequent decline was associated with the liberation of northern parts of the Kharkiv region and the displacement of Russian forces toward the borders, which sharply reduced targeted attacks and led to a temporary reduction in shelling. The second peak in June–July 2022 was driven by renewed shelling in liberated areas, tactical shifts in warfare, and the intensified use of missiles targeting Kharkiv and its surroundings. By 2023, a relative stabilisation occurred, with only sporadic cases observed. Although official data on child evacuations are unavailable, this trend is likely attributed to the large evacuation efforts that occurred in the first months of the war [2, 19].

This study shows a male predominance consistent with prior conflict research [4, 20, 21], though no significant differences in injury patterns by sex or age were found and may stem from the limited sample size. The only statistically significant association calculated in this study was between Face Injury vs. Age 12–17 years (*p* = 0.044). However, the small data set of the study, and inconsistent age categorisation in previous studies, complicates comparisons [4]. This also highlights the need for larger datasets in future studies to clarify demographic influences on injury patterns and whether there is a need for age-specific prevention strategies.

In accordance with the results of this study, previous research has indicated that paediatric and adult injury patterns are broadly similar, often involving the extremities, head, abdomen, and thorax [4]. The younger children of this study were particularly vulnerable to head injuries and burns, likely due to their smaller stature and increased proximity to ground-level heat and blast effects. This finding aligns with studies from Afghanistan, Syria, and Iraq, although conflicts in the Middle East have reported a higher prevalence of pelvic and head injuries [20, 22]. This may be due to the comparatively higher rates of victim-activated improvised explosive devices (IEDs) in Middle Eastern conflicts, which are believed to disproportionately harm children due to their behavioural and physical characteristics (e.g. playing with unfamiliar objects) [4, 23].

The median Injury Severity Score (ISS) was 9, where over one-third of patients sustained severe (ISS > 16) or critical injuries (ISS > 25). Adolescents (12–17 years) suffered the most severe injuries (ISS > 25), possibly due to greater mobility and exposure. This trend may also be attributable to younger children with critical injuries being less likely to survive long enough to reach medical care. Few prior studies on paediatric war-related trauma have included severity metrics such as Injury Severity Score, making comparisons difficult [4], but studies conducted in Iraq and Afghanistan over a 10-year period also reported a median ISS of 9 [24]. Data from Syria, however, have indicated much higher ISS scores, primarily due to critical head injuries [20]. However, those findings may reflect a selection bias, as the Syrian patients with milder injuries were likely treated locally before being transferred to the larger medical centre where the study was conducted.

While over half of the patients achieved good recovery at discharge, many experienced moderate to severe disabilities. As mentioned in the Method, functional outcomes were determined based on clinician-recorded discharge summaries using qualitative descriptors and supported by clinical data. Standardised and validated functional outcome tools could not be applied due to the retrospective nature of the study, hence limiting the comparability of outcomes to other conflict settings.

Reports from Ukrainian clinicians have described cases of early discharge due to targeted attacks on healthcare facilities by Russian forces [10]. Such attacks, in violation of the Geneva Conventions [25], raise concern about the adequacy of post-operative care. Early discharge may hinder full recovery, but it remains unclear whether any patients in this study were prematurely discharged due to such security concerns. Consequently, the observed disability rates may reflect true injury severity or could be inflated due to limited in-hospital recovery time.

Nevertheless, these findings underscore the importance of paediatric rehabilitation and their vulnerability. Ongoing attacks, healthcare infrastructure damage, and displacement of medical professionals have impacted rehabilitation access, especially in eastern Ukraine [26, 27], leaving children with complex injuries without essential long-term support. Addressing the rehabilitation needs, particularly for those with amputations or severe disabilities, is critical for future intervention planning. Clinical systems should prioritise early integration of rehabilitation into acute trauma workflows, and invest in decentralised, mobile, or remote rehabilitation services where conventional care pathways are disrupted.

## Limitations

This study has several limitations that should be considered when interpreting the findings. First, potential selection bias exists due to the study’s focus on hospital-admitted patients, thereby excluding pre-hospital fatalities and those who may have died before receiving medical care. The lack of access to morgue data further limits representation of the most severe injuries, possibly leading to underestimation of overall injury severity and mortality rates. For example, there have been reports of civilian casualties, including children, that have suffered from gunshot wounds, although no such injuries were seen in this cohort [28].

Although clinical data were fully obtained for all included patients, issues with medical documentation completeness were encountered. These stemmed from two primary factors: (1) inconsistent recording practices in medical histories, with some records lacking details on injury mechanisms; and (2) partial loss or damage of documentation due to the hostilities, particularly affecting sections related to injury mechanisms. Despite these challenges, the available records provided sufficient information to meet the study’s objectives.

The study’s definition of war-related injuries focused on direct trauma from explosive mechanisms (e.g., blasts and shelling), potentially underrepresenting the broader impact of conflict-related trauma. Indirect effects, such as burns from unsafe heating, falls due to damaged infrastructure, and road traffic accidents linked to poor road conditions, may also be significant sources of injury but were not captured within this classification. Previous research in Iraq, for example, showed that while bombings comprised 44% of intentional injuries, they represented only 4% of the total injury burden, with indirect consequences contributing far more to overall morbidity [29].

The study reports low rates of pre-existing medical conditions, which may reflect documentation gaps rather than a true absence of comorbidities. Prior evidence suggests a risk of underdiagnosis of chronic conditions such as asthma or type 1 diabetes among Ukrainian children [30].

The study is based on data from a single region, Kharkiv, one of the most heavily impacted areas during the conflict. While this enhances internal consistency, it limits generalisability to other regions in Ukraine that may differ in terms of conflict exposure, healthcare accessibility, and evacuation logistics.

The retrospective design inherently limits the ability to assess long-term outcomes. Functional status at discharge was recorded, but no longitudinal follow-up was conducted, precluding conclusions on extended recovery trajectories or the development of late complications.

Finally, the absence of standardised functional outcome measures at discharge constrains the ability to compare rehabilitation needs and outcomes across settings or with previous conflict studies. Future research would benefit from the development and application of simple, validated functional assessment tools tailored for use in conflict zones and low-resource settings.

## Conclusion

This study provides novel insights into paediatric trauma as a result of modern warfare in a high-income European setting. Key findings include the prominence of blast and shelling injuries, the overall similar injury distribution to previous modern armed conflicts, and that many children are discharged with high rates of disability. These results emphasise the need for comprehensive trauma care that addresses acute injuries and long-term rehabilitation. The findings provide an initial understanding that will be helpful in improving trauma care protocols, resource allocation, and rehabilitation strategies for children affected by the war.

## Data Availability

The underlying data of this study were sensitive and retrieved from patient records. Data from the study database can be made available upon reasonable request, provided that such access complies with the conditions outlined in the ethical approval.
